# Comment on “YouTube as a source of information for restless leg syndrome”

**DOI:** 10.1590/0004-282X-ANP-2020-0544

**Published:** 2021-06-16

**Authors:** Alisha DUGGAL, Taghreed ALWAN, Shan ALI, Tomasz SZMUDA, Paweł SŁONIEWSKI

**Affiliations:** 1 Medical University of Gdansk, Scientific Circle of Neurology and Neurosurgery, Gdansk, Poland. University of Gdansk Medical University of Gdansk Scientific Circle of Neurology and Neurosurgery Gdansk Poland; 2 Medical University of Gdansk, Neurosurgery Department, Gdansk, Poland. University of Gdansk Medical University of Gdansk Neurosurgery Department Gdansk Poland

Dear Editor,

We read with great interest the study by Arikanoglu et al. entitled “YouTube as a source of information for the restless leg syndrome (RLS)” ^1^ . The paper shows that much of medical information on YouTube is low-quality or biased. While the paper offers crucial insight, we believe that the study may have benefited from including more keywords for its analysis.

In their study, one keyword, “restless leg syndrome”, was used to find the videos. We attest that additional terms could have been used, including “restless legs”. This keyword would have widen the search and capture more individuals searching for information about RLS. In a different online study on RLS, Ingram et al. chose to use the additional keyword “restless legs”, rather than only “restless leg syndrome” ^2^ . On Google Trends, the term “restless legs” has a 3-times higher average search volume than “restless leg syndrome” ^3^ . This shows that the public searches more often for the symptom rather than the actual syndrome name.

We used both “restless legs” and “restless leg syndrome” as a search title to analyze the 30 first YouTube videos. The term “restless legs” resulted in 28 medically relevant videos. Eighteen of them (60%) were published by private hospitals, clinics and medical associations, whereas four videos (13%) were published by practitioners and the remaining six videos (20%) by nonmedical specialists. In comparison, the term “restless leg syndrome” resulted in 27 medically relevant videos, one less (3%). Uploads by private hospitals, clinics and medical associations differed by six more videos (20%). By practitioners and nonmedical specialists, there was one video more (4%) and four videos more (13%), respectively. Thus, as we have shown, an online study that fails to take into account the extra term “restless legs” may be limited in its analysis.

Given the change in nomenclature from restless leg syndrome to Willis-Ekbom disease in 2013 by the Restless Leg Syndrome Foundation, we also believe that the keywords “Willis-Ekbom disease” or “Willis-Ekbom” should have been used ^4^ . Although the Foundation changed back to the original name in 2015 ^5^ , journalistic coverage of the name change may have made patients more likely to use the new one.

The evaluation of YouTube content on restless leg syndrome conducted by Arikanoglu et al. provides insight on the reliability of information about this subject on the platform. Our letter highlights some limitations and encourages researchers to use additional keywords to allow for a more robust analysis.
